# Improved cardiorespiratory fitness after occupational rehabilitation in merged diagnostic groups

**DOI:** 10.1186/s40557-018-0227-y

**Published:** 2018-02-28

**Authors:** Anne Lovise Nordstoga, Paul Jarle Mork, Marius Steiro Fimland

**Affiliations:** 1 Department of Public Health and General Practice, Faculty of Medicine, NTNU, Norwegian University of Science and Technology, 7491 Trondheim, Norway; 2 Hysnes Rehabilitation Center, St. Olavs Hospital, Trondheim University Hospital, Trondheim, Norway

**Keywords:** Acceptance and commitment therapy, Exercise, Mental disorders, Musculoskeletal disease

## Abstract

**Background:**

Various occupational inpatient rehabilitation programs are established in Norway. This study aimed to assess change in cardiorespiratory fitness, pain, anxiety, depression, and quality of life in persons on long-term sick leave due to musculoskeletal-, mental or unspecific disorders after participation in multicomponent inpatient occupational rehabilitation.

**Methods:**

Twenty-five women and five men (mean age 45.2 years, SD 6.7, range 30–57) volunteered to participate in the study. The participants attended either 8 or 17 full days of occupational multicomponent rehabilitation including physical exercise, cognitive behavioral therapy in the form of acceptance and commitment therapy (ACT), and development of a tailored plan for return to work. Cardiorespiratory fitness was assessed by the Åstrand/Ryhming cycle test at the start and end of rehabilitation program, and at one-year follow-up. Changes in somatic and mental health were measured by questionnaires up to 4 months after start of the program.

**Results:**

Linear mixed models showed that the maximal oxygen uptake increased by 1.1 mL°kg-1°min^− 1^ during the rehabilitation program and by 3.7 mL°kg-1°min^− 1^ at one-year follow-up. There were minor improvements in somatic and mental health, and quality of life.

**Conclusions:**

This study indicates that occupational inpatient multicomponent rehabilitation including physical exercise and ACT may promote a long-term increase in physical exercise that is sufficient to induce a significant increase in cardiorespiratory fitness.

**Trial registration:**

The current study is not registered, but is part of a larger trial registered at clinicaltrials.gov (No.: NCT01926574, registered 21. Aug 2013).

## Background

Long-term sickness absence may have negative consequences for individuals and their families, employers, as well as the general society [1]. Norway has the highest level of sickness absence in Europe, with musculoskeletal- and mental disorders being the two most common diagnostic groups. To improve workability and increase return to work rates, various occupational rehabilitation programs are provided both by public and private sector stakeholders.

Although most of the patients on long-term sick leave are treated in primary care, comprehensive tertiary institutional care occupational rehabilitation programs have existed for more than 30 years in Norway. Physical exercise most often constitute a considerable part of such programs due to its documented benefits on several health-related outcomes – which in turn could lead to improved workability and work participation [2]. Regular physical exercise is associated with better musculoskeletal- and mental health [3, 4], and physical functioning [5]. Furthermore, physical exercise is associated with reduced risk of sickness absence and disability pension [2, 6, 7], although not all studies report such associations [8]. The physical exercise program in the current inpatient rehabilitation setting aimed to increase cardiovascular and muscular fitness. Patient education aimed at promoting sustainable physical exercise habits, improving knowledge about physical exercise and possible health benefits, and reducing fear of movement was an integrated part of the physical exercise program.

Cognitive behavioral therapy approaches are also commonly employed in occupational rehabilitation programs. In the present study acceptance and commitment therapy (ACT) was used, which emphasizes accepting both negative and positive experiences, while using a person’s values to guide them towards their goals [9]. Some studies have suggested that ACT may reduce chronic pain [10], depression [11, 12], and anxiety [11, 13]. Persons participating in ACT must themselves decide what their values are. This can for instance be to improve health and fitness, which may necessitate a change in physical exercise habits.

The main aim of the study was to assess acute and long-term changes in cardiorespiratory fitness following participation in a multi-component occupational rehabilitation program. A second aim was to assess change in pain, anxiety, depression, and quality of life. All participants in the study were on long term sick leave due to musculoskeletal-, mental or unspecific disorders and were invited to take part in an inpatient multicomponent occupational rehabilitation program.

## Methods

### Participants and recruitment

The study sample consisted of 30 patients (25 women, 5 men) participating in one of two (16 persons in the short program and 14 persons in the long program) multicomponent rehabilitation programs carried out at Hysnes Rehabilitation Center, Norway, in the period from August 2013 to February 2014. The inclusion criteria were: 1) age 18–60 years, 2) sick leave duration 2–12 months with a current sick leave status of at least 50%, and 3) an ICPC-2 (International Classification of Primary Care, 2nd ed.) diagnosis within the L (musculoskeletal), P (psychiatric) or A (unspecific disorders) categories. Furthermore, a physician and a physiotherapist determined eligibility in screening sessions based on the the following exclusion criteria: 1) alcohol or drug abuse, 2) serious somatic (e.g. cancer, heart disease) or psychological disorder (e.g. suicide attempts, psychosis, ongoing manic episode), 3) a specific disorder requiring specialized treatment, 4) pregnancy, 5) currently participating in another treatment program, 6) insufficient comprehension of Norwegian language to participate in group sessions and to fill out questionnaires, 7) scheduled for surgery within the next 6 months, and 8) serious problems with functioning in a group setting. Characteristics of the study participants are presented in Table 1.

**Table 1:** Baseline characteristics of the baseline study sample (*n* = 30) and the participants that participated at one-year follow-up test of maximal oxygen uptake and those who did not. Values are mean ± SD

	Baseline study sample (*n* = 30)	Not participating at one-year follow-up (*n* = 20)	Participating at one-year follow-up (*n* = 10)	P*
Men/Women	5/25	2/18	3/7	
Age (years)	45.2 ± 6.7	44.7 ± 6.9	46.3 ± 6.5	0.533
BMI (kg/m^2^)	27.8 ± 5.6	28.0 ± 5.5	27.5 ± 5.9	0.804
Maximal oxygen uptake	25.1 ± 8.5	24.3 ± 6.1	32.0 ± 10.5	0.021
High physical demands at work, n				
Not at all/to a small extent	10	7	3	
Somewhat/to a large extent	17	10	7	
HADS anxiety (0–21)	7.4 ± 4.4	7.1 ± 4.6	7.9 ± 4.0	0.637
HADS depression (0–21)	5.7 ± 4.1	6.0 ± 4.4	5.2 ± 3.6	0.632
Quality of life (0–1)	0.80 ± 0.10	0.79 ± 0.11	0.82 ± 0.08	0.531
Average pain last week (0–10)	4.2 ± 1.8	4.8 ± 1.5	3.1 ± 1.9	0.016
ICPC-2 diagnoses, n				
Musculoskeletal	13	11	2	
Mental	12	6	6	
Unspecific	2	0	2	

Note: for three persons questionnaire data and diagnosis data was not available

*Abbreviations:* Body mass index, BMI; Hospital anxiety and depression scale, HADS; International Classification of Primary Care, 2nd ed., ICPC-2

*Independent samples t-test comparison of the two sub-samples not participating at one-year follow-up vs. participating at one-year follow-up

Eligible participants were informed about the study at the day of arrival at the rehabilitation center and were given the opportunity to sign up for the study. All of the eligible participants at the rehabilitation center accepted the invitation to participate in the study. The study was approved by the Regional Committee for Ethics in medical research (no.: 2012/1241) and all participants signed an informed consent before enrollment. The study was carried out according to the latest revision of the Declaration of Helsinki.

### Rehabilitation programs

The occupational multicomponent rehabilitation program at Hysnes Rehabilitation Center have been described in detail elsewhere [14]. In brief, participants in this study participated in either a “long” or a “short” multicomponent inpatient program. Both programs included both individual and group-based activities organized as a 6–7 h workday at the rehabilitation center. Each group activity included a maximum of eight participants. The long program lasted ~ 3.5 work weeks (17 days) and the short program lasted 4 + 4 days separated by 2 weeks where participants lived at home. In both programs, 2–3 designated coordinators per group were involved in coordinating and implementing the programs. ACT based group discussions were led by team coordinators. The coordinators who mentored the participants were supervised by a certified ACT instructor before and during (monthly) the intervention. In addition, each participant had 2 individual meetings with the coordinator in the short program and 5 individual meetings with the coordinator in the long program, to discuss and get advice on work-related problem-solving and create a plan for return to work. Each participant developed a personalized plan for physical exercise in cooperation with their coordinator and the designated exercise coach. Exercise sessions at the rehabilitation center were both individual and group-based. The scheduled physical exercise performed in the long program consisted in total of 12 h of indoor sessions (including strength training and endurance training), with each session lasting 1–1.5 h. Additionally, 8 h of outdoor activities were included during the stay. The physical exercise in the short program consisted in total of 10.5 h (including both strength training and endurance training), with each session lasting 1–1.5 h.

### Outcomes

Cardiorespiratory fitness was assessed by the Åstrand/Ryhming cycle test on a cycle ergometer (828 E Monark, Sweden). A heart rate (HR) monitor (Polar T31, Polar Electro, Finland) with a chest strap transmitter was used to record HR. The participant was instructed to maintain a cadence of 60 rpm throughout the test. The starting workload was estimated by taking age, gender and training status into consideration. If the initial workload was set too high or too low, it could be adjusted during the test, although, the final workload was continued for 6 min. The test was approved if the participant achieved a HR between 120 and 160 bpm after 6 min. Maximal oxygen uptake (mL, kg^− 1^, min^− 1^) was estimated by taking the average of the two last measurements of the HR, and applying this value to the Åstrand/Ryhming nomogram [15], corrected by the Åstrand age factor [16, 17]. Measuring HR at submaximal loads and extrapolating them into the expected age-adjusted maximal (HR) values is commonly used as an indirect measurement of maximal oxygen uptake [17].

Anxiety and depression were assessed with The Hospital Anxiety and Depression Scale (HADS) [18]. It consists of 14 items scored on a 4-point Likert scale according to intensity of symptoms the last week. The maximum score is 21 on both subscales for anxiety and depression, respectively. HADS is widely used and has been found to perform well in assessing severity of symptoms and to detect anxiety and depression. A cut-off score of 8 has been shown to give an optimal balance between sensitivity and specificity on both subscales [19]. HADS was answered at the start and end of the rehabilitation program, and 4 months after starting the program.

To assess pain we used one item from the Brief Pain Inventory (BPI): ‘please rate your pain by circling the one number that best describes your pain on the average’ The participants were asked to grade the average pain during the last week on a 0 (no pain) to 10 (worst imaginable pain) numeric rating scale [20]. The pain question was answered at the start and end of the rehabilitation program, and 4 months after starting the program.

Health-related quality of life was assessed with 15D [21]. It contains 15 dimensions covering physical, mental and social well-being and generates a total score ranging from 1 (no problem on any dimension) to 0 (being dead). It has been suggested that the generic minimal important change is ±0.015 and a large change is ±0.035 [22].

### Data collection

Objective measurements (height, weight and cardiorespiratory fitness) were collected at enrolment (baseline) and after completing the rehabilitation program (post-test), and took place at the rehabilitation center. A 1 year follow-up assessment of cardiorespiratory fitness was performed at St. Olavs Hospital in Trondheim, 12–14 months after the baseline test.

Internet-based questionnaires were used to collect information about health-related outcomes. The participants received text messages on their mobile when it was time to answer questionnaires and as reminders if they did not respond. For the 1 year follow up measurement of cardiorespiratory fitness, we sent an invitation letter to all 30 participants. Fourteen replied and of these, 10 patients were willing to participate at the follow-up test in October 2014 (five from the short program and five from the long program).

### Statistical analyses

The statistical analyses were performed using Stata for Windows, version 13.1 (StataCorp LP, College Station, Texas). Multilevel mixed-effects linear regression was used to assess change in cardiorespiratory fitness from before to after the rehabilitation program, and at the one-year follow-up. Pain, anxiety, depression and quality of life was assessed with the same test before, after and up to 4 months after the start of the programs. The Shapiro-Wilk tests was used to explore normality. Cardiorespiratory fitness was not normally distributed and these values were therefore log-transformed. Test-time was included as a fixed effect and person as a random effect. In a secondary analysis of complete cases, a Wilcoxon matched-pairs signed rank test was also used to assess change in cardiorespiratory fitness from the start to the one-year follow-up. A *P*-value of 0.05 was considered to be significant. Values are reported with 95% confidence intervals (CI). An independent samples t-tests was used to compare the baseline characteristics of the participants who participated at the one-year follow-up test of cardiorespiratory fitness with the participants that did not participate at the follow-up.

## Results

Table 1 presents the baseline characteristics of the participants. All 30 participants enrolled in the study performed the post-test of maximal oxygen uptake immediately after completing the rehabilitation program. Ten of the 30 participants took part in the one-year follow-up of maximal oxygen uptake (five participants in the long program and five participants in the short program). These participants had similar baseline characteristics compared to the participants that did not participate in the one-year follow-up, except for pain and maximal oxygen uptake (Table 1).

Twenty-seven participants answered the questionnaires at baseline and post-test, while 20 participants answered the questionnaires at baseline, post-test and at the 4 month follow-up.

**Fig. 1:**
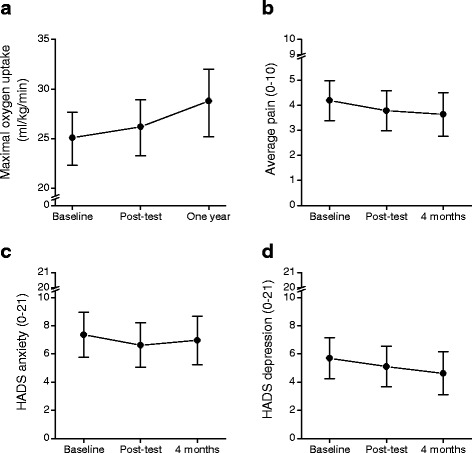
**a** Maximal oxygen uptake (ml/kg/min), **b** average pain (0–10), **c** HADS depression score (0–21) and **d** HADS anxiety score (0–21) at baseline, end of rehabilitation program, and at one-year follow-up, estimated by mixed models. Values are mean. Error bars indicate 95% confidence intervals

Multilevel mixed-effects linear regression showed that the maximal oxygen uptake (Fig. 1) increased significantly from 25.1 ml/kg/min (95% CI 22.5–27.9) at baseline to 26.2 (95% CI 23.5–29.1; *P* = 0.031) after the rehabilitation program and to 28.8 (95% CI 25.6–32.4; *P* < 0.001) at one-year follow-up. When considering only persons with complete data, the maximal oxygen uptake increased from 31.9 (95% CI 23.9–40.0) at baseline to 36.9 (95% CI 27.3–46.4) at the one-year follow-up (Wilcoxon test, *P* < 0.01). Compared to baseline, average pain last week, anxiety, depression, and quality of life did not significantly change at post-test and 4 months follow-up (*P* ≥ 0.086 for all comparisons).

## Discussion

The current study evaluated the short- and long-term changes in cardiorespiratory fitness, musculoskeletal pain, psychological symptoms, and quality of life in persons with musculoskeletal-, mental or unspecific disorders taking part in an occupational multicomponent rehabilitation program. The results showed significant improvement in maximal oxygen uptake from before to after rehabilitation and with further improvements after 1 year. Only minor and non-significant improvements were observed for musculoskeletal pain, psychological symptoms, and quality of life from before to after completion of the rehabilitation program and at 4 months follow-up.

A significant increase in the maximal oxygen uptake of 1.1 ml/kg/min was found from baseline to post-test, despite that the period between the tests was only 3 weeks. Similar results have been found after short training interventions in healthy persons [23, 24], but these studies employed training with very high intensity. However, improvement in cardiovascular fitness was recently found in fibromyalgia patients performing a 12 week intervention with moderate intensity exercise [25], and a review by Mannerkorpi and Henriksson [26] found that exercise at 55–90% of maximum HR improved cardiovascular fitness in patients with chronic widespread musculoskeletal pain. Further, a possible reason why the patients in our study improved after such a short training period with less intensive training could be the very low cardiorespiratory fitness at baseline. One could expect a rapid increase in maximal oxygen uptake due to enhanced amount of exercise.

Interestingly, the maximal oxygen uptake also continued to increase during the follow-up period after completion of the rehabilitation program. The substantial 14% improvement at the one-year follow-up compared to baseline may suggest that the multicomponent rehabilitation program induced sustainable changes in the physical activity levels of these patients. However, a possible reason for the significant improvement in long-term cardiovascular fitness may be a potential attrition bias in the study. The baseline score on maximal oxygen uptake was significantly lower in the initial study sample compared to the group who performed the one-year follow-up. Patients with higher maximal oxygen uptake may be more interested in a healthy lifestyle including physical activity, and more motivated to sustain the level of physical activity over time.

Although there was no control group in this study, a large reference material shows that the expected age-related reduction in maximal oxygen uptake is close to 1% per year [27]. Regarding the physical exercise components of the interventions, they aimed to improve endurance and strength capacity and knowledge about physical activity, as well as promoting sustainable physical activity. We therefore believe that the improvement more likely is due to the intervention than other factors. A personalized plan was developed during the program in cooperation with their coordinator and a designated exercise coach. The exercise program was based on clinical judgment as well as the goals and interests of the patient. The progress was evaluated during the rehabilitation stay, leading into a training program that the patient was encouraged to perform after the end of the rehabilitation program. Studies of ACT to promote an increase in physical activity are scarce, however; some studies recruiting healthy subjects have showed positive results [28, 29]. A pilot study by Butryn and colleagues [28] investigating whether ACT promotes short-term increase in physical activity in healthy females, reported that ACT increased the level of physical activity more than an educational intervention during 8 weeks follow-up. In contrast, a randomized controlled trial recruiting physically inactive adults did not find any difference between the experimental group receiving ACT and a control group in change of objectively or subjectively measured physical activity levels for 3 and 6 months follow-up. However, the study found differences between the groups in change of cognitions related to exercise and physical activity, in favor of the ACT group [29].

The 3.7 ml/min/kg improvement in maximal oxygen uptake in the participants in this study may have important health benefits. In a recent meta-analysis [30] a dose-response analysis of cardiorespiratory fitness as predictor of all-cause mortality and cardiovascular disease and coronary heart disease were performed. They found that an increase of 3.5 ml/min/kg in cardiorespiratory fitness reduced the risk of all-cause mortality and cardiovascular- and coronary heart disease by 13% and 15%, respectively.

The somatic and mental health, and quality of life were not significantly improved; however, it should be noted that these analysis were based on a small study sample and thereby limited statistical power. Recently, a larger study of patients with musculoskeletal, psychological or unspecified disorders enrolled at the same rehabilitation center reported improved quality of life from baseline to 12 months, while only marginal changes were found for pain, anxiety and depression [31]. Still, in a recent meta-analysis of acceptance- and mindfulness-based interventions for chronic pain [32], small but significant short- and long-term improvements were found for pain, depression, and anxiety.

There are some limitations regarding the present study that should be taken in consideration. This was a small, non-controlled study including 30 participants at baseline, and only 10 participants at the one-year follow-up cycle test. However, we used mixed models which uses all available data. This model-based approach reduced the magnitude of the change from baseline to the one-year follow-up compared to the secondary analysis of complete cases (increment of 3.7 vs. 5.0 ml/min/kg). Although there were some differences, most variables were similar between those who attended and did not attend the one-year follow-up. This was not the case for maximal oxygen uptake and pain, which could indicate an attrition bias. Another limitation is that the measurement of cardiorespiratory fitness was an indirect measure of maximum oxygen uptake by the Åstrand/Ryhming cycle test, which may lead to uncertainty compared to direct measurements. A known uncertainty is systematic underestimation of maximum oxygen uptake in unfit individuals [16]. However, this is related to heart rate measurements, variation in work efficiency and maximum heart rate, which are mostly constant within the same individual. The test is reliable with respect to detecting changes in cardiorespiratory fitness [16]. To evaluate within-subject changes, the Åstrand/Ryhming cycle test has been suggested to be a useful measurement, and the reliability is confirmed by several studies [33, 34], which was also illustrated in this study where a small but statistically significant 1.1 ml/kg/min improvement could be observed from baseline after completion of the rehabilitation program. Moreover, although we suggest that the rehabilitation program may be responsible for the increase in cardiorespiratory fitness, we have no information on occupational physical activity in the follow-up period, which potentially could contribute to an increased activity level. However, occupational physical activity does not seem to have the required intensity to improve cardiorespiratory fitness [35]. Finally, the study sample consisted mainly of women (83%), which generally have lower cardiorespiratory fitness than men [27]. However, changes in cardiorespiratory fitness would primarily reflect an altered exercise volume and intensity for both genders.

## Conclusion

The results of this study show short-term and long-term increases in maximum oxygen uptake in persons with musculoskeletal-, psychological, or unspecific disorders after completion of an occupational inpatient multicomponent rehabilitation program. This suggests that an intensive rehabilitation period, including exercise in combination with acceptance and commitment therapy, can induce meaningful and sustainable improvements in cardiorespiratory fitness. Randomized controlled trials should validate these findings.

## Abbreviations

ACT: Acceptance and commitment therapy
BPI: Brief Pain Inventory
HADS: The Hospital Anxiety and Depression Scale
HR: Heart rate
